# Supplementary material for: Advanced Nanotechnology-Based Nucleic Acid Medicines
Source: Pharmaceutics. 2024 Oct 25;16(11):1367. doi: 10.3390/pharmaceutics16111367 (PMC11597528; doi:10.3390/pharmaceutics16111367)
Supplement: Supplementary file 1 [file pharmaceutics-16-01367-s001.zip › pharmaceutics-3130385-supplementary.pdf]

Table S1. List of ongoing clinical trials of LNP medicines (Oct 2024)

	Drug code	Company	Disease	Molecule	NCT number	Phase	Status	Last update posted
1	NTLA-2002	Intellia Therapeutics	Hereditary Angioedema	guideRNA and mRNA	NCT06634420	III	Recruiting	2024/10/15
2	ARCT-810	Arcturus Therapeutics, Inc.	OTC Deficiency丨Ornithine Transcarbamylase Deficiency丨OTCD	mRNA	NCT06488313	II	Recruiting	2024/7/5
3	BP1001	Bio-Path Holdings, Inc.	AML	ASO	NCT02781883	II	Recruiting	2023/3/28
4	mRNA-3745	ModernaTX, Inc.	Glycogen Storage Disease	mRNA	NCT05095727	I/II	Recruiting	2024/8/12
5	BEAM-302	Beam Therapeutics Inc.	Alpha 1-Antitrypsin Deficiency	guideRNA and mRNA	NCT06389877	I/II	Recruiting	2024/6/24
6	STX-001	Strand Therapeutics Inc.	Advanced Solid Tumor	mRNA	NCT06249048	I/II	Recruiting	2024/8/22
7	quaratusugene ozeplasmid	Genprex, Inc.	Carcinoma, Non-Small Cell Lung	pDNA	NCT04486833	I/II	Recruiting	2024/6/26
8	quaratusugene ozeplasmid	Genprex, Inc.	Small Cell Lung Cancer Extensive Stage	pDNA	NCT05703971	I/II	Recruiting	2024/5/20
9	OTX-2002	Omega Therapeutics	Hepatocellular Carcinoma and Other Solid Tumor Types Known for Association with the MYC Oncogene	mRNA	NCT05497453	I/II	Recruiting	2024/9/25
10	VRC-FLUNPF099-00-VP (H1ssF_3928)	National Institute of Allergy and Infectious Diseases (NIAID)	Influenza	mRNA	NCT05755620	I	Recruiting	2024/9/27
11	DCVC H1 HA mRNA vaccine	National Institute of Allergy and Infectious Diseases (NIAID)	Influenza	mRNA	NCT05945485	I	Recruiting	2024/9/27
12	ARCT-032	Arcturus Therapeutics, Inc.	Cystic Fibrosis	mRNA	NCT05712538	I	Recruiting	2023/12/4
13	TI-0010	National Drug Clinical Trial Institute of the Second Affiliated Hospital of Bengbu Medical College	COVID-19丨COVID-19 Immunisation	mRNA	NCT06205524	I	Recruiting	2024/1/18
14	MT-302	Myeloid Therapeutics	Epithelial Tumors, Malignant	mRNA	NCT05969041	I	Recruiting	2024/1/18

Table S2. List of clinical trials of nanotechnology-based nucleic acid medicine outside Japan in past to present (Oct-2024)

#	Drug code	Disease	Nanotechnology	Molecule	Target	Company	NCT Number	Phase	Status
Lipid-based development									
1	ALN-VSP02	Solid tumors	LNP	siRNA	KSP and VEGF	Alnylam Pharmaceuticals	NCT00882180	I	Completed
							NCT01158079	I	Completed
2	ALN-PCS02	Hypercholesterolemia	LNP	siRNA	PCSK9	Alnylam Pharmaceuticals	NCT01437059	I	Completed
3	ALN-TTR01	TransThyRetin (TTR)-mediated amyloidosis	LNP	siRNA	TTR	Alnylam Pharmaceuticals	NCT01148953	I	Completed
4	TKM-ApoB	Liver cancer	LNP, SNALP(stable nucleic acid-lipid particles)	siRNA	Apo B	Tekmira Pharmaceuticals	NCT00927459	I	Terminated
5	TKM-080301(TKM-PLK1)	Liver cancer	LNP, SNALP	siRNA	PLK1	Tekmira Pharmaceuticals	NCT01437007	I	Completed
6	TKM-100201(KM-Ebola)	Ebola Virus Infection	LNP, SNALP	two types of siRNAs	VP24, VP35, l-polymerase	Tekmira Pharmaceuticals	NCT01518881	I	Terminated
7	TKM-100802	Ebola Virus Infection	LNP, SNALP	two types of siRNAs	VP24, VP35, l-polymerase	Tekmira Pharmaceuticals	NCT02041715	I	Terminated
8	ALN-TTR02(Patisiran)	TransThyRetin (TTR)-mediated amyloidosis	LNP, MC3	siRNA	TTR-mediated amyloidosis (ATTR)	Alnylam Pharmaceuticals	NCT 01559077	I	Completed
							NCT 01617967	II	Completed
							NCT 01961921	II	Completed
							NCT 01960348	III	Completed
							NCT02939820	Approved for marketing	
							NCT02510261	III	Completed
							NCT03862807	III	Completed
9	ND-L02-s0201	Fibrosis	LNP	siRNA	HSP47	Nitto Denko Corporation	NCT 01858935	I	Completed
							NCT02227459	I	Completed
							NCT03241264	I	Completed
							NCT03538301	II	Completed
10	ARB-001467	Hepatitis B, Chronic	LNP	siRNA	HBV proteins	Arbutus Biopharma	NCT02631096	II	Completed
11	DCR-MYC	Solid Tumors, Multiple Myeloma, Non-Hodgkins Lymphoma	LNP	siRNA	MYC	Dicerna Pharmaceuticals	NCT 02110563	I	Terminated
		Liver cancer	LNP	siRNA	MYC		NCT02314052	I/II	Terminated
12	LErafAON-ETU	Neoplasms	Liposome	ASO	C-Raf	INSYS Therapeutics Inc	NCT00100672	I	Completed
13	MRX34-101	primary liver cancer or other selected solid tumors or hematologic malignancies	Liposome	miR-34a mimic	Oncogenes, including FOXP1, BCL2, HDAC1, and CTNNB1	Mirna Therapeutics, Inc.	NCT01829971	I	Terminated
14	BP1001	hematological malignancies	Liposome	ASO	Grb2	Bio-Path Holdings, Inc.	NCT01159028	I	Completed
15	DCR-PH1	Primary hyperoxaluria type 1	LNP	siRNA	Glycolate oxidase	Dicerna Pharmaceuticals, Inc., a Novo Nordisk company	NCT02795325	I	Terminated
16	siRNA-EphA2-DOPC	Advanced cancers	Liposome	siRNA	EPHA2	M.D. Anderson Cancer Center	NCT01591356	I	Active, not recr

									uiting
17	MTL-CEBPA(MTL-501)	Liver cancer	Liposome(SMARTICLES)	small activating RNA	CEBPA	Mina Alpha Limited	NCT04105335	I	Active, not recruiting
18	NBF-006	Non-Small Cell Lung, Pancreatic, or Colorectal Cancer	Liposome	SiRNA	GSTP	Nitto BioPharma, Inc.	NCT03819387	I	Completed
19	Atu027	Advanced solid cancers	Lipoplex, AtuPLEX®	siRNA	PKN3	Silence Therapeutics	NCT00938574	I	Completed
							NCT01808638	I/II	Completed
							NCT02191878	I/II	Completed
		Neuroendocrine Tumors (NET) and Adrenocortical Carcinoma (ACC)					NCT01262235	I/II	Completed
							NCT04710641	II	Active, not recruiting
							NCT03319849	III	Completed
Synthetic polymer-based development									
20	CALAA-01	Solid Tumors	cationic cyclo-dextrin based polymer (CDP)	siRNA	RRM2	Arrowhead (Calando Pharmaceuticals)	NCT00689065	I	Terminated
21	siG12D LODER	Pancreatic cancer	LODER polymer(PLGA)	siRNA	KRAS G12D	silenseed	NCT01188785	I	Completed
							NCT01676259	II	Unknown status
22	MK-4621	Advanced/Metastatic or Recurrent Solid Tumors	JetPEI™	CpG oligonucleotide	RIG-1 activation	Merck Sharp & Dohme LLC	NCT03065023	I/II	Terminated
							NCT03739138	I	Terminated
23	STP705	Hypertrophic scar	Histidine–Lysine copolymers (polypeptide NP)	siRNA	TGF-β 1and Cox-2	Sirnaomics	NCT02956317	I/II	Completed
		Advanced solid tumors		siRNA	PTGS2 gene inhibitor; TGFB1 gene inhibitor	Sirnaomics	NCT05196373	I/II	Not yet recruiting
24	STP707	solid tumor	Histidine–Lysine copolymers(HKP+H)	siRNA	TGF-β1 and COX-2	Sirnaomics	NCT05037149	I	Active, not recruiting
Biomaterial-based development									
25	TargomiR	Malignant Pleural Mesothelioma	Nonliving bacterial minicells(EDV)	miR-16 mimic	Oncogenes, including BCL2, CDK1 and JUN	Asbestos Diseases Research Foundation	NCT02369198	I	Completed
26	exoASO-STAT6(CDK-004)	Advanced Hepatocellular Carcinoma (HCC) and Patients with Liver Metastases from EIther Primary Gastric Cancer or Colorectal Cancer (CRC)	Exosome	ASO	STAT-6	Codiak BioSciences Inc	NCT05375604	I	Terminated
27	iExosomes	Pancreatic cancer	mesenchymal stem cell (MSC)-derived exosomes	siRNA	KRAS G12D	M.D. Anderson Cancer Center	NCT03608631	I	Active, not recruiting
28	TTX-MC138	Advanced Solid Tumors	Dextran-coated iron oxide NP	Antagomirs (miR-10b inhibitors)		TransCode Therapeutics	NCT05908773	I	Completed
						TransCode Therapeutics	NCT06260774	I/II	Recruiting
29	NU-0129	Recurrent Glioblastoma or Gliosarcoma Undergoing Surgery	Gold NP decorated with OEG/PEG copolymer	siRNA	BCL2L12 gene inhibitor	Diakonos Oncology Corp	NCT03020017	I	Completed
